# Visuo–Vestibular Virtual Reality-Based Training for People with Stroke: A Feasibility Study

**DOI:** 10.3390/healthcare14050625

**Published:** 2026-03-02

**Authors:** Jacopo Piermaria, Diego Piatti, Sara De Angelis, Gianluca Paolocci, Matteo Marucci, Roberta Annicchiarico, Viviana Betti, Susan L. Whitney, Marco Tramontano

**Affiliations:** 1 Santa Lucia Foundation, Scientific Institute for Research and Health Care, 00179 Rome, Italy; jacopo.piermaria@gmail.com (J.P.); s.deangelis@hsantalucia.it (S.D.A.); 2 Laboratory of Neuromotor Physiology, IRCCS Santa Lucia Foundation, 00179 Rome, Italy; d.piatti@hsantalucia.it (D.P.); gia.paolocci@gmail.com (G.P.); 3 Department of Psychology, Sapienza University of Rome, 00185 Rome, Italy; m.marucci91@gmail.com (M.M.); viviana.betti@uniroma1.it (V.B.); 4 Laboratory of Neuroscience and Applied Technology, Santa Lucia Foundation IRCCS, 00179 Rome, Italy; 5 Clinical and Behavioral Neurology Laboratory, IRCCS Fondazione Santa Lucia, 00179 Rome, Italy; roberta.annicchiarico@aslroma1.it; 6 UOC Geriatria, ASL Roma 1, 00139 Rome, Italy; 7 Departments of Physical Therapy and Otolaryngology, University of Pittsburgh, Pittsburgh, PA 15260, USA; whitney@pitt.edu; 8 Translational Rehabilitation Sciences Group, Department of Biomedical and Neuromotor Sciences (DIBINEM), Alma Mater Studiorum University of Bologna, Via Zamboni, 33, 40126 Bologna, Italy; 9 Unit of Occupational Medicine, IRCCS Azienda Ospedaliero-Universitaria di Bologna, Via Giuseppe Masserenti, 9, 40138 Bologna, Italy

**Keywords:** stroke, vestibular physical therapy, vestibular rehabilitation, virtual reality

## Abstract

**Background/Objectives**: Stroke frequently leads to balance deficits. Vestibular physical therapy (VPT) may enhance postural control through neuroplastic mechanisms. Virtual reality (VR) can provide ecologically valid environments for rehabilitation, increasing patient engagement. **Methods**: In this randomized feasibility study, nine individuals with chronic stroke were randomized to either a Real visuo–vestibular rehabilitation group (*n* = 6) or a Sham VR group (*n* = 3) to explore the feasibility of the protocol and randomization procedures rather than to compare clinical efficacy. Both groups were trained in immersive VR environments for 12 sessions. The Real group experienced visuo–vestibular stimuli requiring sensorimotor integration; the Sham group trained in the same environments without such stimuli. Feasibility was assessed through attendance, participation (Pittsburgh Rehabilitation Participation Scale, PRPS), and user satisfaction (USEQ). Safety and acceptability were monitored through adverse event reporting. Secondary exploratory outcomes included measures of balance—the Mini Balance Evaluation Systems Test (MiniBESTest), the Berg Balance Scale (BBS), and the Performance-Oriented Mobility Assessment (POMA)—as well as functional independence (Barthel Index), health-related quality of life (Stroke-Specific Quality of Life Scale, SSQoL), and a set of spatiotemporal and gait quality parameters derived from inertial measurement unit (IMU) data collected during the 10-Meter Walk Test and the Figure of 8 Walk Test. **Results**: All participants completed the protocol without adverse events. Participation, as measured by the PRPS, remained consistently high across sessions (mean ≥5.7/6), while USEQ scores indicated excellent user satisfaction (mean ≥28/30). Exploratory analyses revealed improvements in MiniBESTest and BBS scores for the Real group. Instrumental measures derived from IMUs demonstrated improvements across groups. **Conclusions**: Exploratory outcomes suggested positive trends in balance improvements, and the integration of clinical scales with wearable sensors proved feasible and informative.

## 1. Introduction

Stroke is the second leading cause of death worldwide and remains one of the leading contributors to long-term disability [1]. Although incidence rates have declined in many high-income countries, a worrying trend has emerged in younger populations, where both prevalence and incidence are increasing [2,3,4]. At the onset of stroke, approximately 50% of survivors are unable to walk, and residual gait and balance deficits persist in nearly half of patients [5]. Critically, only 30–50% of stroke survivors achieve community ambulation, which represents a key milestone for participation and autonomy in daily life [5,6]. These enduring impairments highlight the urgent need for innovative and effective rehabilitation approaches.

Restoring dynamic balance and gait during the subacute and chronic phases of stroke is essential to reduce fall risk, improve independence, and ultimately enhance quality of life [7,8]. Among available interventions, vestibular physical therapy (VPT) has shown promising effects in improving balance and mobility in people with stroke [9,10,11]. This approach is thought to stimulate neural reorganization, promote the strengthening of neural networks, and enhance postural control mechanisms [10,12]. Importantly, VPT differs from generic vestibular rehabilitation because it emphasizes exercise-based strategies specifically designed to enhance neuroplasticity, rather than relying on pharmacological or compensatory interventions [13].

The effectiveness of VPT underscores the importance of individualized and tailored rehabilitation strategies, based on careful patient assessment, to address the heterogeneity of gait and balance deficits after stroke [14]. At the same time, advances in technology are offering new possibilities to deliver such interventions. In particular, virtual reality (VR) has emerged as a promising adjunct to conventional rehabilitation. VR allows the creation of safe, controlled, and ecologically valid environments in which patients can engage in tasks that closely resemble real-life challenges. Evidence suggests that VR may enhance patient engagement, foster motivation, and improve the functional transfer of training gains, with reported benefits for balance, upper limb function, and activity participation [11,15,16].

Bringing these two lines of evidence together, the integration of VPT principles with immersive VR environments represents a novel and innovative rehabilitation paradigm that has not been sufficiently explored in people with chronic stroke yet. Building on this rationale, we developed Visuo–Vestibular Virtual Reality-based training (VVR), a fully immersive rehabilitation program designed to train visuo–vestibular interaction. The protocol achieves this through exposure to ecologically unpredictable environments and spontaneous head movements.

We hypothesized that VVR would be safe, feasible, and well accepted by people with stroke. Furthermore, we expected that the combination of clinical and instrumental assessments would accurately detect and quantify changes in balance and gait performance. Therefore, the primary aim of this feasibility study was to evaluate the safety, feasibility, and acceptability of VVR in individuals with chronic stroke while also assessing the viability and integration in the context of stroke rehabilitation of the integrated clinical and instrumental assessment framework.

## 2. Materials and Methods

### 2.1. Participants

Participants with chronic stroke, more than six months after the event, were recruited through the Rehabilitation Services of the Santa Lucia Foundation Institute for Research and Healthcare. Eligible participants were between 18 and 70 years of age and had a Functional Ambulation Category (FAC) score of at least three, indicating the ability to walk with some degree of independence. Patients were excluded if they presented with comorbidities that could interfere with balance or gait, such as pre-existing vestibular disorders, severe orthopaedic conditions, sensory deficits caused by conditions such as diabetes, peripheral neuropathies, and patients with severe visual and visual field impairments, as well as those with significant cognitive impairment (Mini-Mental State Examination score < 24) or a diagnosis of epilepsy.

This study was approved by the local Ethics Committee (Register No. 171/SL/24). All procedures were conducted in accordance with the Declaration of Helsinki and institutional guidelines. This study is registered at ClinicalTrials.gov (NCT06780488). All participants provided written informed consent before enrolment.

### 2.2. Development and Configuration of the Virtual Reality Environment

The immersive virtual reality (VR) environments were developed using Unity 3D (https://unity.com) and deployed on a Meta Quest Pro headset. The virtual scenarios, including ecological contexts such as a sidewalk and a marketplace, were specifically designed by Myndek (www.myndek.com) to support the visuo–vestibular interaction training protocol described above. To allow participants to walk freely and naturally, the guardian boundary system of the headset was temporarily disabled under controlled and supervised conditions, enabling full-body movement within a dedicated 30 × 17 m motion capture area. This configuration ensured that participants could explore the virtual environments without interruptions or artificial spatial constraints. Before each session, a calibration procedure was performed to ensure spatial correspondence between the real and virtual environments, preventing any mismatch or potential collisions with physical boundaries. Additionally, in the market scenario, hand tracking was enabled to capture hand movements and allow direct manipulation of virtual objects without handheld controllers, promoting a more naturalistic and embodied interaction. All walking sessions in VR were continuously supervised by an experimenter positioned beside the participant, who ensured safety and provided verbal guidance when necessary. This setup allowed participants to move independently in the VR space while maintaining a high level of experimental control and safety.

### 2.3. Protocol and Randomization

This pilot feasibility study adopted a randomized controlled design. Participants were randomly assigned using block randomization (block size of 3). To ensure allocation concealment, an external researcher not involved in the recruitment or clinical assessment generated the allocation sequence and placed the assignments in sequentially numbered, opaque, sealed envelopes. Both groups trained in immersive VR environments developed within the VVR project.

**Figure 1:**
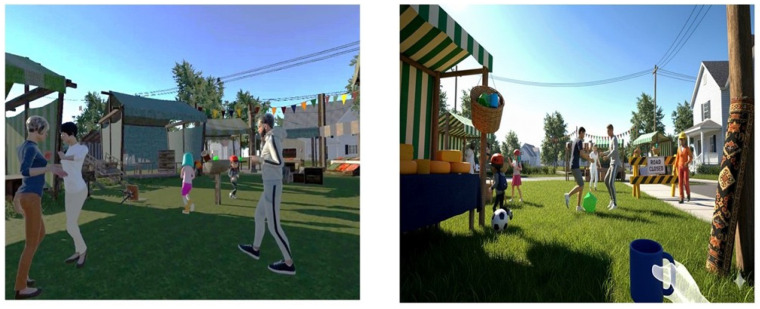
Immersive virtual reality environments used for the Real Visuo–Vestibular Rehabilitation group. Both contexts incorporated dynamic visuo–vestibular stimuli and unexpected events to enhance balance and gait training.

The Real VVR group engaged in tasks specifically designed to challenge visuo–vestibular interaction, such as navigating ecological contexts like a street or a marketplace where sudden and unpredictable visual events occurred (Figure 1).

These visual stimuli were intentionally designed to appear at the periphery of the patient’s field of view, thereby necessitating non-voluntary or unplanned rapid head movements to direct the gaze towards the unexpected target (Supplementary Material Video S1). This configuration, as demonstrated in the video, causes quick motions that stimulate vestibular response, eliciting natural vestibular activation and promoting the integration of both visual and vestibular feedback. The training aligns with the concept that active and passive head movements produce distinct sensory reafferences within the vestibular nuclei and cerebellum, contributing differently to motor control and sensory prediction [17,18]. In the Sham condition, participants walked in the same virtual environments as the Real VVR group as an active control group; however, the visuo–vestibular component of the intervention was intentionally inactivated. The VR scenarios were presented as predictable, without any of the visual motion cues, dynamic scene changes, or unexpected sensory perturbations used to elicit visuo–vestibular responses. While all patients were aware of the technology being used, they were not informed of the specific differences between the Real and Sham stimuli protocols.

The intervention for both groups consisted of 20 min sessions three times per week for four consecutive weeks, delivered through the Meta Quest Pro headset (Meta Platforms, Inc., Menlo Park, CA, USA). The exercise program was supervised by physiotherapists with more than five years of experience in neurorehabilitation.

The VVR was provided in addition to each participant’s conventional rehabilitation program, which included three weekly sessions (~40 min each) focused on gait and upper-limb function. All participants in both the Real and Sham groups received a similar dosage of standard neurorehabilitation. Sessions were individually tailored to each patient’s clinical presentation and functional goals, including stretching exercises, muscle strengthening exercises, and gait training exercises.

### 2.4. Feasibility Outcomes

Trained physiotherapists who were blind to the group assignment conducted clinical and instrumental evaluations to guarantee the validity of the exploratory data. Participants underwent assessments at baseline (T0), at the end of treatment (T1), and at one-month follow-up (T2). The primary feasibility outcomes were attendance and completion rates; participation was measured with the Pittsburgh Rehabilitation Participation Scale (PRPS) [19], and user satisfaction was measured with the User Satisfaction Evaluation Questionnaire (USEQ) [20].

### 2.5. Secondary Exploratory Outcomes

Secondary exploratory outcomes included clinical measures of balance, independence, and quality of life (MiniBESTest, Berg Balance Scale, POMA, Barthel Index, and Stroke-Specific Quality of Life Scale).

### 2.6. Instrumental Assessment

The IMU-based gait assessments were performed by two physiotherapists specifically trained in gait analysis with inertial sensors. The recordings were performed during straight walking using the 10-Meter Walk Test (10MWT) [21] and during curved-path walking using the Figure of 8 Walk Test (F8WT).

**Figure 2:**
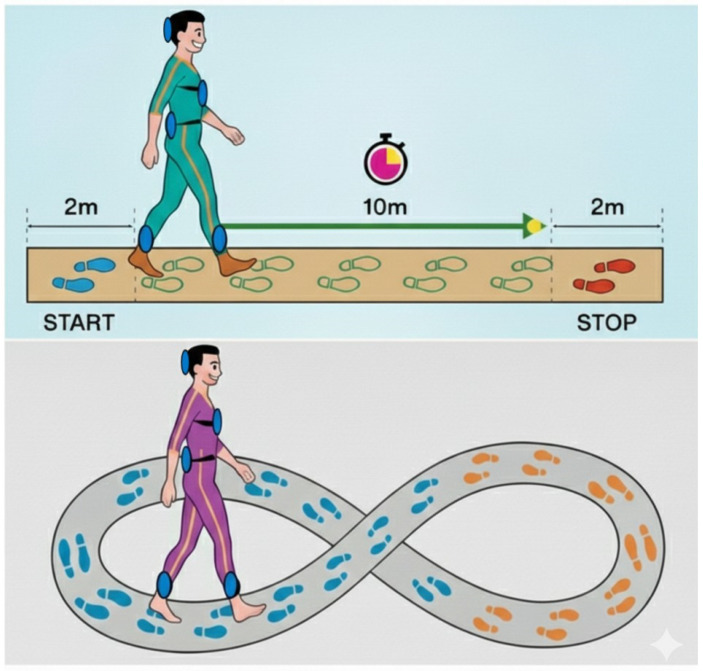
Sensor-based assessment during the 10MWT and the F8WT.

During the performance of the assessment, participants were equipped with five synchronized IMUs (128 Hz, Opal, APDM, Portland, OR, USA), measuring three-dimensional linear accelerations and angular velocities. IMUs were located on the occipital cranium bone, near the lambdoid suture of the head (H), at the center of the sternum (S), and at the L4/L5 level, just above the pelvis (P). For step and stride segmentation, one IMU was placed on each shank, just above the lateral malleoli. All IMUs were attached to the participant’s body with Velcro straps (Figure 2).

The data were processed in the Matlab^®^ environment (MATLAB R2022b, MathWorks, Natick, MA, USA) for the extraction of spatiotemporal and gait quality parameters. The following spatiotemporal parameters were investigated: average walking speed (WS), average stride frequency (Freqstride), stride length, and number of strides. The step segmentation relied on a peak detection algorithm on the medial–lateral (ML) angular velocity signals measured by the two IMUs on the shanks [22,23,24]. Moreover, postural stability and symmetry of gait parameters were evaluated using data from IMUs placed on the sacrum, trunk, and head, as described in a previous research [25].

### 2.7. Adverse Events

Adverse events were systematically monitored throughout the intervention to ensure participant safety. Before each session, participants were asked about new or ongoing symptoms. During sessions, trained staff continuously observed participants for signs of discomfort, dizziness, nausea, pain, or other unexpected responses, documenting any incidents immediately. After each session, participants reported any delayed symptoms.

### 2.8. Statistical Analysis

Given the exploratory nature of this study, statistical analyses were conducted to describe trends rather than to confirm efficacy. The normality of distributions was verified with the Shapiro–Wilk test. Within-group and between-group comparisons were performed using non-parametric tests, including the Wilcoxon and Mann–Whitney tests, while repeated-measures nonparametric analyses (nparLD, F1–LD–F1 design) were applied to sensor-based indices. ANOVA-type statistics and Relative Treatment Effects (RTEs) were reported to provide further insight, but results were interpreted cautiously due to the small sample size and feasibility scope.

## 3. Results

### 3.1. Participants

**Figure 3:**
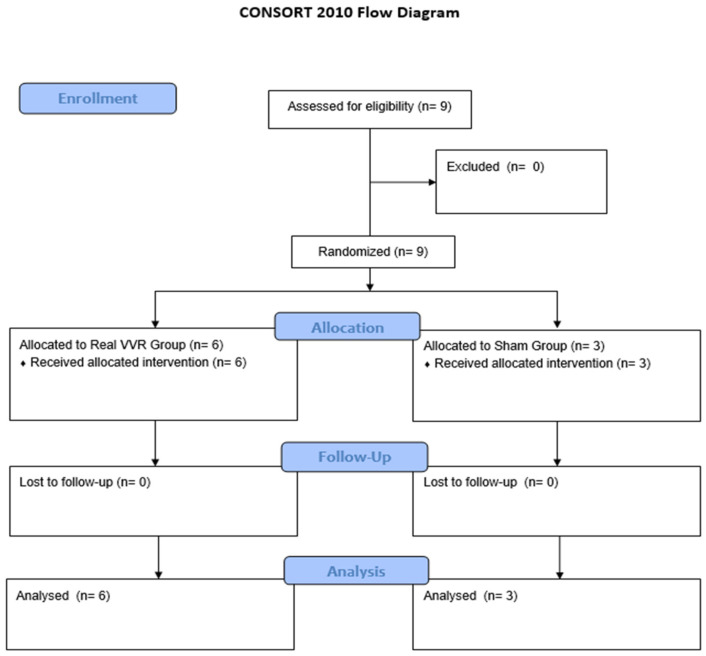
CONSORT flow diagram illustrating participant progression through this study.

Nine participants, eight men and one woman, with a mean age of 61.6 years (SD ± 8.5), were included in this study. Six participants were randomized to the Real visuo–vestibular rehabilitation group (mean age 63.83 years, SD ± 5.95; five males, one female; four haemorrhagic stroke, two ischemic stroke), and three participants were assigned to the control group (mean age 56 years, SD ± 13; three males; all three with haemorrhagic stroke) (Figure 3). The sample size for this randomized feasibility study was determined based on the primary objective of evaluating protocol safety and acceptability rather than clinical efficacy.

The following results are presented descriptively to illustrate temporal trends. Demographic and clinical characteristics are reported in Table 1.

### 3.2. Participation and User Satisfaction

**Figure 4:**
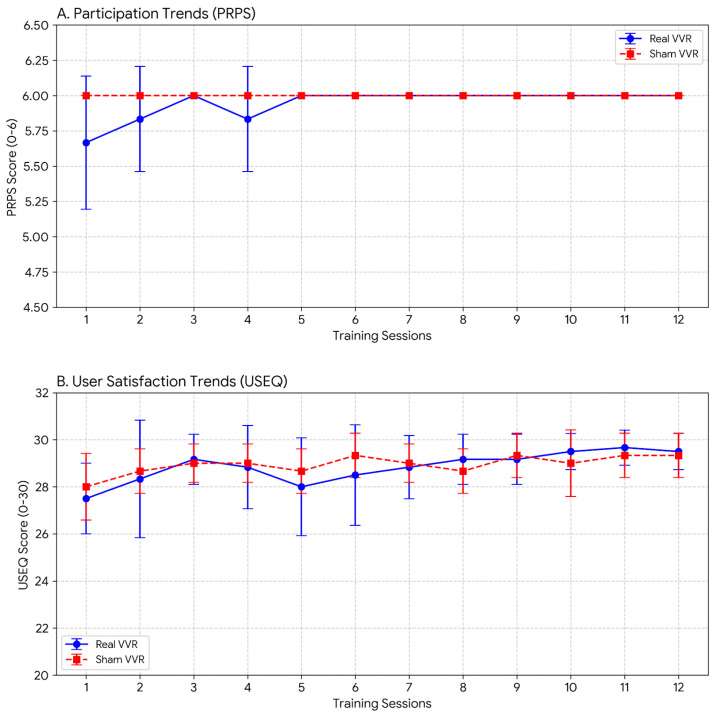
Primary feasibility outcomes across the 12 training sessions. VVR = Visuo–Vestibular Virtual Reality-based training.

Participation in training sessions, as measured by the PRPS, remained consistently high in both groups, with average scores approaching the maximum possible value across all training sessions. For each session, a value between 0 and 6 was obtained, with higher scores indicating greater participation. The complete results are presented in the Supplementary Material (S1 and S2). User satisfaction, captured by the USEQ, also reached very high levels, with mean scores close to the upper limit of the scale, suggesting excellent acceptance of the immersive VR system. Question 5 is reverse-scored, reflecting discomfort, while all other questions contribute positively to the total score. The USEQ score ranged from 0 to 30. A total score of 30 represents the maximum degree of user satisfaction with the virtual rehabilitation system. The individual and mean scores for each USEQ question, along with internal consistency metrics, are presented as Supplementary Material (S1). A summary of the primary feasibility outcomes is presented in Table 2 and Figure 4.

### 3.3. Analyses of Secondary Exploratory Outcomes

**Figure 5:**
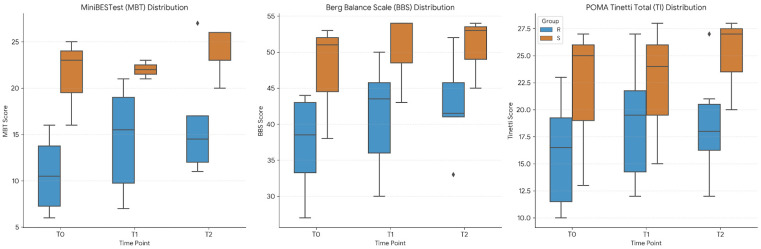
Distribution of secondary clinical outcome scores. Boxplots represent the scores for the Real group (Real, blue) and the control group (Sham, orange). MBT: MiniBESTest; BBS: Berg Balance Scale; POMA: Performance-Oriented Mobility Assessment. ♦ indicates a statistical outlier.

Exploratory analyses of the MiniBESTest scores showed improvement in the Real VVR group from baseline to both post-treatment and follow-up, with no further change between T1 and T2. A similar pattern was observed for the Berg Balance Scale, with improvements from baseline to post-treatment and follow-up, while scores remained stable thereafter. The Sham group did not show changes in clinical scale scores across the assessments. For POMA, the Real group showed an improvement from T0 to T1. No differences were detected in the Sham group (Figure 5).

**Figure 6:**
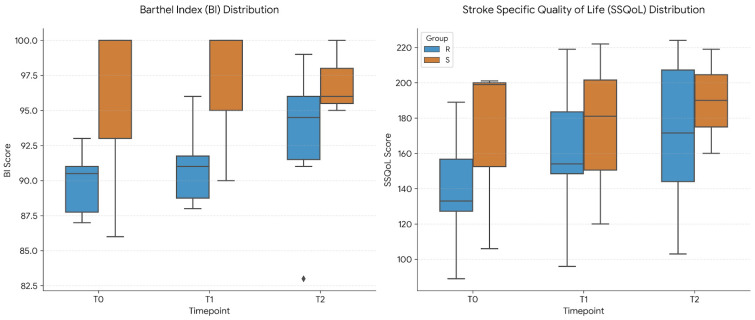
Distribution of secondary outcome scores. Boxplots represent the scores for the Real group (Real, blue) and the control group (Sham, orange). BI: Barthel Index; SSQoL: Stroke-Specific Quality of Life Scale. ♦ indicates a statistical outlier.

Both groups did not show differences in the Barthel Index scores. Analysis of SSQoL scores revealed an effect of time for the Real group across the three timepoints (Figure 6).

The 10MWT data did not show any notable trend in the Sham group. In the Real VVR group (*n* = 6), the post-intervention measurements showed higher values in stride frequency, stride length, and walking speed compared to pre-intervention. In addition, improvements were observed in the postural stability of the pelvis and head in the AP axis, as well as in the ML axis for the pelvis. Post-intervention values of smoothness of gait (LDLJ) in the CC and AP axes also improved with respect to the pre-intervention values.

Similarly, the F8WT data showed no notable trends in the Control group. In the Real VVR group, post-intervention measurements indicated a shorter test duration and higher values of stride frequency and smoothness of gait in the ML and CC axes compared with pre-intervention. The most detailed results are provided in the Supplementary Material (S3).

Currently, no adverse events have been reported in the data collected during the feasibility study.

## 4. Discussion

The present feasibility study investigated a novel rehabilitation paradigm that combines principles of VPT with immersive VR environments, focusing specifically on the retraining of visuo–vestibular interaction in people with chronic stroke. All participants completed the intervention without adverse events, and both participation and satisfaction levels were consistently high throughout the four weeks of treatment. These findings indicate that VVR training is safe, well accepted, and feasible to implement in a clinical setting. These preliminary results are aligned with previous research that provides evidence that immersive visuo–vestibular rehabilitation can be successfully delivered using commercially available VR technology and integrated within conventional neurorehabilitation programs, enabling targeted training on unpredictable saccadic movements on a broader range of oculomotor strategies parameters [26]. To comprehensively evaluate the impact of these mechanisms, the next randomized controlled trial will incorporate advanced eye-movement assessments. In future studies, this approach will allow objective quantification of parameters such as velocity, latency, amplitude, and accuracy, providing deeper insight into how visuo–vestibular rehabilitation may drive neural reorganization and adaptation within vestibulo-cerebellar pathways.

The high adherence and satisfaction observed are particularly important because they suggest that patients not only tolerated the intervention but also engaged actively with the training. Engagement is a crucial determinant of rehabilitation outcomes, and immersive VR offers opportunities to increase motivation by providing realistic and interactive environments [27]. In our study, patients reported a high degree of enjoyment and comfort during training sessions, which may support long-term adherence if the intervention were to be extended or integrated into routine practice. These findings are consistent with previous reports that immersive VR can enhance ecological validity and patient participation in rehabilitation [11,15]

Although this study was not powered to test efficacy, exploratory outcomes suggest that patients in the Real visuo–vestibular rehabilitation group experienced improvements in balance, aligning with prior evidence that showed that VPT enhances postural stability and gait control after stroke [10,12,14]. Importantly, the visuo–vestibular challenges embedded within immersive environments may have triggered spontaneous and non-programmed head movements in response to sudden or unpredictable visual perturbations. Although our study is exploratory in nature, it is reasonable to hypothesize that such movements are known to activate vestibular reafference mechanisms. This sensory feedback, generated by self-initiated actions, plays a critical role in helping the nervous system distinguish internally generated motion from externally imposed movement [17,18,28].

Wearable inertial sensors allowed for the quantification of postural stability, symmetry, and smoothness of gait during functional tasks, offering a level of sensitivity beyond traditional clinical scales.

Several limitations should be acknowledged. The unequal distribution of participants across groups restricts the generalizability of the findings and limits the interpretation of group differences. A potential ceiling effect may have been introduced due to the relatively high baseline functional status of the sample. Also, this high starting point limits our results’ generalizability. The feasibility design precludes conclusions about efficacy, and the promising trends observed must be confirmed in larger randomized controlled trials. Another limitation is the confounding effect of concurrent conventional rehabilitation, which may have contributed to the overall improvements observed in both groups. While this reflects real-world clinical practice, it means that the specific therapeutic effect of VVR cannot be fully isolated from the benefits of standard neurorehabilitation. Consequently, the positive trends and improvements observed in balance and gait must be interpreted with caution, and future studies are needed. In addition, the visuo–vestibular stimuli were standardized for all participants, which may not fully address individual variability in training needs. Future developments should aim to personalize VR scenarios to the characteristics of each patient, possibly through adaptive algorithms or co-design processes involving clinicians and engineers. Furthermore, a further consideration involves the potential therapeutic effect of immersive VR within the Sham condition. Although the visuo–vestibular stimuli were inactivated for this group, the mere act of walking within an immersive, high-fidelity virtual environment may still promote balance and gait improvements. Lastly, we need to recognize the possible impact of expectancy effects. Compared to traditional therapy, immersive VR is frequently seen as a “cutting-edge” intervention, which could raise treatment expectations. Future research should use standardized questionnaires to formally quantify and control for participant expectations, even though our Sham group used the same equipment to reduce this bias. Furthermore, for future studies, it will be useful to include specific questionnaires to improve the monitoring of possible minor adverse events. Overall, while the positive trends observed in the assessed parameters are encouraging, they should be regarded as preliminary signals only, given the exploratory nature of this study and the potential confounding effect of concomitant conventional rehabilitation.

## 5. Conclusions

Exploratory outcomes suggested positive trends in balance improvements, and the integration of clinical scales with wearable sensors proved feasible and informative. Given that this study is only exploratory in nature, future studies should investigate the clinical effectiveness of immersive visuo–vestibular training and explore the personalization of VR scenarios to better match patients’ individual needs.

## Figures and Tables

**Table 1:** Characteristics of participants included in this feasibility study. Data are presented as means with standard deviations. The range (minimum and maximum values) is represented in square brackets [Min–Max]. VVR = Visuo–Vestibular Virtual Reality-based training.

Characteristic	Total (*n* = 9)	Real VVR Group (*n* = 6)	Sham VVR Group (*n* = 3)
Mean age (SD)	61.6 ± 8.5 [52–68]	63.83 ± 5.95 [52–68]	56 ± 13 [41–63]
Sex (M/F)	8/1	5/1	3/0
Type of stroke	6 haemorrhagic, 2 ischemic	3 haemorrhagic, 2 ischemic, 1 not reported	3 haemorrhagic
Barthel Index (mean ± SD)	91.67 ± 5.24 [86–100]	89.83 ± 2.40 [87–93]	95.33 ± 8.08 [86–100]
Time since stroke (months)	13 ± 7.46 [8–30]	14 ± 9.5 [11–30]	11 ± 2.88 [8–14]

**Table 2:** Summary of primary feasibility outcomes. Results are presented as mean (SD).

Group	PRPS	USEQ
Real	5.93 (0.12)	28.82 (1.25)
Sham	6 (0.0)	28.90 (0.92)

## Data Availability

The original contributions presented in this study are included in the article and/or Supplementary Materials. Further inquiries can be directed to the corresponding author.

## Supplementary Materials

The following supporting information can be downloaded at: https://www.mdpi.com/article/10.3390/healthcare14050625/s1, Video S1: Virtual reality training video. Material S1: Pittsburgh Rehabilitation Participation Scale (PRPS—feasibility outcome) scores for individual participants; Material S2: USEQ (feasibility outcome) scores for individual participant; Material S3: Additional tables on the results of the statistical analysis of inertial sensors data collected during the 10 Meters Walking Test (10MTWT) and Figure of 8 Walking Test (F8WT); Table S1: Results of statistical analysis for spatiotemporal, postural stability and smoothness of movement indices obtained from 10MWT data; Table S2: Results of statistical analysis for spatiotemporal, postural stability and smoothness of movement indices obtained from F8WT data.

## Abbreviations

The following abbreviations are used in this manuscript:

VPT	Vestibular Physical Therapy
VR	Virtual Reality
VVR	Visuo–Vestibular Virtual Reality-based training
PRPS	Pittsburgh Rehabilitation Participation Scale
USEQ	User Satisfaction Evaluation Questionnaire
