# Supplementary material for: Visuo–Vestibular Virtual Reality-Based Training for People with Stroke: A Feasibility Study
Source: Healthcare (Basel). 2026 Mar 2;14(5):625. doi: 10.3390/healthcare14050625 (PMC12984202; doi:10.3390/healthcare14050625)
Supplement: Supplementary file 1 [file healthcare-14-00625-s001.zip › healthcare-4129172-supplementary.pdf]

		Training sessions												
Subject	*VRR Group*	*1*	*2*	*3*	*4*	*5*	*6*	*7*	*8*	*9*	*10*	*11*	*12*	*Score*
1	Real	6	6	6	6	6	6	6	6	6	6	6	6	6
2	Real	5	5	6	5	6	6	6	6	6	6	6	6	5.7
3	Real	6	6	6	6	6	6	6	6	6	6	6	6	6
4	Real	6	6	6	6	6	6	6	6	6	6	6	6	6
5	Real	5	6	6	6	6	6	6	6	6	6	6	6	5.9
6	Real	6	6	6	6	6	6	6	6	6	6	6	6	6
7	Sham	6	6	6	6	6	6	6	6	6	6	6	6	6
8	Sham	6	6	6	6	6	6	6	6	6	6	6	6	6
9	Sham	6	6	6	6	6	6	6	6	6	6	6	6	6

Supplementary Material (S1): Pittsburgh Rehabilitation Participation Scale (PRPS—feasibility outcome) scores for individual participants (indicated by rows) within the Real (R) and Sham (S) groups across the rehabilitation sessions (indicated by the different columns). Scores range from 0 to 6, with higher values indicating greater participation in the therapy sessions.

		Individual training session scores for the USEQ for each subject over the 12 sessions and the mean score for all sessions												*Score*
Subject	*VVR Group*	*1*	*2*	*3*	*4*	*5*	*6*	*7*	*8*	*9*	*10*	*11*	*12*	
1	Real	28	30	29	30	24	26	29	29	29	30	30	30	28.6
2	Real	25	23	27	25	27	25	26	27	27	28	28	29	26.4
3	Real	27	28	30	29	29	30	30	30	30	30	30	30	29.4
4	Real	28	30	30	30	30	30	29	30	30	30	30	30	29.7
5	Real	27	29	29	29	28	30	29	29	29	29	30	28	28.8
6	Real	30	30	30	30	30	30	30	30	30	30	30	30	30
7	Sham	29	30	30	30	30	30	30	30	30	30	30	30	29.9
8	Sham	26	28	28	28	28	30	29	28	30	30	30	30	28.7
9	Sham	29	28	29	29	28	28	28	28	28	27	28	28	28.1

Supplementary Material (S2): USEQ (feasibility outcome) scores for individual participants within the Real (R) and Sham (S) groups across the rehabilitation sessions. Scores range from 0 to 30.

Supplementary material (S3): Additional tables on the results of the statistical analysis of inertial sensor data collected during the 10-Meter Walking Test (10MTWT) and Figure of 8 Walking Test (F8WT).4

Given the exploratory nature of this study and the limited sample size, statistical analyses were focused on identifying trends and providing a robust description of the observed changes. Below, we report the results for parameters that reached statistical significance.

For each parameter, the manuscript presents descriptive statistics (mean ± SD) at T0, T1, and T2, alongside change scores (Δ) representing the pre-to-post training magnitude of improvement. To account for the non-parametric distribution of the data and the longitudinal design, ANOVA-type statistics (ATS) were employed using the nparLD framework. ATS is a distribution-free approach that provides a robust alternative to traditional repeated measures ANOVA, particularly suitable for small cohorts, as it does not require multivariate normality or sphericity.
Furthermore, we report the Relative Treatment Effects (RTEs) as an estimation of effect size. In this non-parametric context, the RTE represents the probability that a randomly selected observation from a specific time point or group is larger than an observation randomly selected from the entire dataset. An RTE of 0.50 indicates the absence of an effect, while values deviating from this threshold quantify the direction and strength of the observed trends.

Parameter	Group	T0	T1	T2	Δ (T1 –T0)	p (Group)	p (Time)	p (Group*Time)	RTE Group	RTE Time T0	RTE Time T1	RTE Time T2	RTE Group * T0	RTE Group * T1	RTE Group * T2
**$Freq_{stride}$**	EXP	0.4 ± 0.23	0.5 ± 0.28	0.49 ± 0.29	+0.1	0.37	<0.001	0.73	0,44	0.46	0.59	0.53	0,39	0,50	0,44
	CTR	0.53 ± 0.2	0.61 ± 0.26	0.55 ± 0.24	+0.08				0,61				0,54	0,69	0,61
**Stride Length**	EXP	0.49 ± 0.19	0.72 ± 0.25	0.75 ± 0.31	+0.23	0.11	<0.001	0.71	0.40	0.38	0.63	0.63	0.24	0.47	0.50
	CTR	0.71 ± 0.26	1.11 ± 0.39	1.09 ± 0.29	+0.40				0.69				0.52	0.79	0.77
**WS**	EXP	0.23 ± 0.23	0.4 ± 0.37	0.43 ± 0.43	+0.17	0.31	<0.001	0.84	0.43	0.40	0.60	060	0.31	0.49	0.50
	CTR	0.41 ± 0.25	0.74 ± 0.47	0.65 ± 0.39	+0.33				0.64				0.50	0.71	0.70
**nRMS AP pelvis**	EXP	1.58 ± 0.58	1.33 ± 0.5	1.43 ± 0.61	-0.25	0.26	0.03	0.90	0.57	0.58	0.39	0.42	0.68	0.49	0.54
	CTR	1.19 ± 0.54	0.97 ± 0.6	1.02 ± 0.42	-0.22				0.36				0.47	0.29	0.30
**nRMS ML pelvis**	EXP	1.23 ± 0.31	1.06 ± 0.32	1.16 ± 0.4	-0.17	0.72	0.003	0.88	0.53	0.57	0.42	0.47	0.60	0.46	0.52
	CTR	1.18 ± 0.55	0.99 ± 0.37	1.04 ± 0.47	-0.19				0.44				0.54	0.38	0.42
**nRMS ML trunk**	EXP	1.46 ± 0.52	1.16 ± 0.26	1.21 ± 0.35	-0.30	0.05	0.35	0.33	0.61	0.5	0.43	0.40	0.71	0.54	0.57
	CTR	0.82 ± 0.27	0.83 ± 0.51	0.74 ± 0.26	+0.01				0.28				0.29	0.33	0.23
**nRMS AP head**	EXP	1.78 ± 0.66	1.19 ± 0.47	1.58 ± 0.79	-0.59	0.22	0.01	0.53	0.59	0.56	0.37	0.45	0.68	0.47	0.60
	CTR	1.13 ± 0.77	0.92 ± 0.69	0.94 ± 0.68	-0.21				0.33				0.43	0.26	0.29
**nRMS ML head**	EXP	1.37 ± 0.39	1.07 ± 0.36	1.28 ± 0.41	-0.30	0.14	<0.001	0.27	0.59	0.58	0.34	0.44	0.67	0.48	0.61
	CTR	1.05 ± 0.44	0.75 ± 0.38	0.85 ± 0.27	-0.30				0.33				0.5	0.20	0.28
**LDLJa CC**	EXP	-5.86 ± 0.69	-5.37 ± 0.6	-5.54 ± 0.59	+0.49	0.86	0.02	0.14	0.49	0.44	0.59	0.48	0.37	0.57	0.52
	CTR	-5.55 ± 0.65	-5.35 ± 0.56	-5.64 ± 0.76	+0.20				0.52				0.51	0.61	0.45
**LDLJw AP**	EXP	-4.87 ± 0.44	-4.97 ± 0.6	-4.69 ± 0.52	-0.10	0.02	0.32	0.30	0.39	0.56	0.51	0.60	0.38	0.31	0.48
	CTR	-4.43 ± 0.1	-4.35 ± 0.3	-4.29 ± 0.44	+0.08				0.72				0.73	0.71	0.71

S3_Table S1 – results of statistical analysis for spatiotemporal, postural stability, and smoothness of movement indices obtained from 10MWT data.

Abbreviations: WS: Walking Speed, nRMS: normalized Root Mean Square, LDLJa: Log Dimensionless Jerk of linear acceleration, LDLJw: Log Dimensionless Jerk of angular velocity, AP: antero-posterior, ML: medio-lateral, CC: cranio-caudal, RTE: Relative Treatment Effect.

Parameter	Group	T0	T1	T2	Δ (T1 –T0)	p (Group)	p (Time)	p (Group*Time)	RTE Group	RTE Time T0	RTE Time T1	RTE Time T2	RTE Group * T0	RTE Group * T1	RTE Group * T2
**Duration**	EXP	66.86 ± 42.12	52.83 ± 31.44	54.19 ± 36.02	-14.03	0.33	<0.001	0.48	0.57	0.56	0.41	0.42	0.65	0.53	0.52
	CTR	40.8 ± 39.58	30.63 ± 30.33	29.65 ± 25.48	-10.17				0.36				0.48	0.29	0.33
**Freq$_{stride}$**	EXP	0.4 ± 0.22	0.47 ± 0.25	0.47 ± 0.27	+0.07	0.46	<0.001	0.01	0.45	0.47	0.53	0.57	0.38	0.50	0.49
	CTR	0.49 ± 0.19	0.48 ± 0.2	0.54 ± 0.22	-0.01				0.59				0.56	0.56	0.65
**WS**	EXP	0.26 ± 0.21	0.32 ± 0.28	0.35 ± 0.33	+0.06	0.39	0.01	0.76	0.44	0.46	0.54	0.59	0.37	0.46	0.48
	CTR	0.44 ± 0.28	0.47 ± 0.3	0.53 ± 0.31	+0.03				0.62				0.55	0.62	0.70
**nRMS ML pelvis**	EXP	1.3 ± 0.36	1.17 ± 0.34	1.17 ± 0.42	-0.13	0.65	0.02	0.62	0.53	0.58	0.44	0.43	0.60	0.51	0.49
	CTR	1.25 ± 0.38	1.04 ± 0.46	1.04 ± 0.34	-0.22				0.43				0.56	0.38	0.36
**nRMS ML trunk**	EXP	1.58 ± 0.46	1.52 ± 0.49	1.34 ± 0.31	-0.06	0.02	0.29	0.76	0.62	0.47	0.45	0.40	0.67	0.63	0.56
	CTR	0.99 ± 0.18	0.91 ± 0.44	0.88 ± 0.26	-0.08				0.26				0.28	0.28	0.23
**nRMS ML head**	EXP	1.37 ± 0.33	1.34 ± 0.51	1.28 ± 0.27	-0.03	0.19	0.01	0.75	0.58	0.54	0.39	0.44	0.65	0.53	0.57
	CTR	1.17 ± 0.38	0.87 ± 0.34	0.99 ± 0.23	-0.30				0.33				0.44	0.25	0.30
**LDLJa ML**	EXP	-5.98 ± 0.59	-5.77 ± 0.51	-5.69 ± 0.53	+0.22	0.86	0.05	0.31	0.49	0.42	0.56	0.54	0.40	0.51	0.56
	CTR	-5.83 ± 0.31	-5.29 ± 1.07	-5.78 ± 0.41	+0.54				0.52				0.44	0.62	.51
**LDLJw CC**	EXP	-4.39 ± 0.59	-4.03 ± 0.61	-4.14 ± 0.7	+0.35	0.20	0.04	0.38	0.43	0.46	0.63	0.52	0.33	0.49	0.46
	CTR	-3.88 ± 0.25	-3.37 ± 0.83	-3.84 ± 0.39	+0.51				0.64				0.59	0.76	0.59

S3_Table S2 – results of statistical analysis for spatiotemporal, postural stability, and smoothness of movement indices obtained from F8WT data.

Abbreviations: WS: Walking Speed, nRMS: normalized Root Mean Square, LDLJa: Log Dimensionless Jerk of linear acceleration, LDLJw: Log Dimensionless Jerk of angular velocity, ML: medio-lateral, CC: cranio-caudal, RTE: Relative Treatment Effect
